# Supplementary material for: Genome-Wide Identification of LOX Gene Family and Its Expression Analysis under Abiotic Stress in Potato (Solanum tuberosum L.)
Source: Int J Mol Sci. 2024 Mar 20;25(6):3487. doi: 10.3390/ijms25063487 (PMC10970340; doi:10.3390/ijms25063487)
Supplement: Supplementary file 1 [file ijms-25-03487-s001.zip › Figure S1.pptx]

## Slide 1
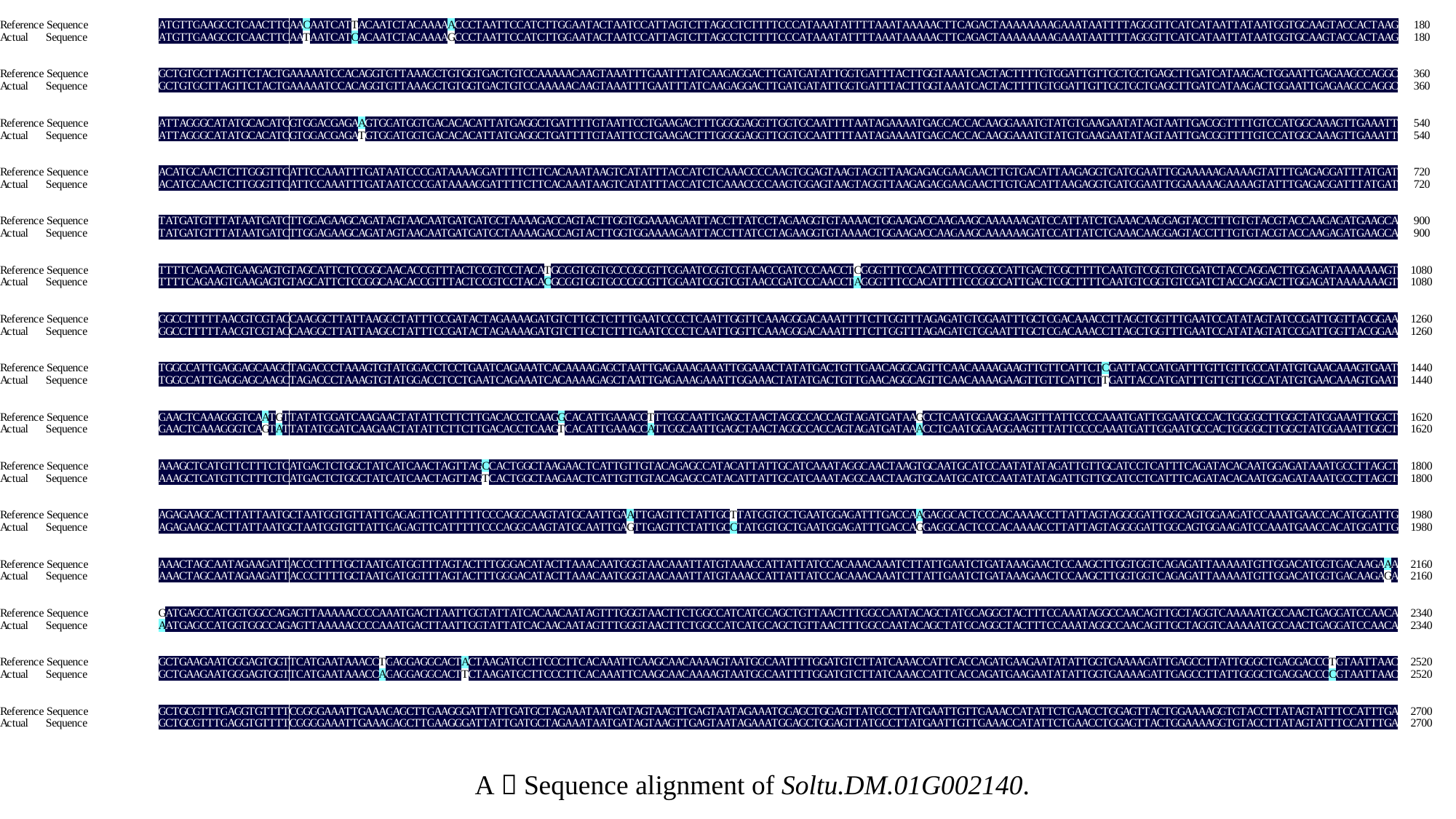

A：Sequence alignment of Soltu.DM.01G002140.

## Slide 2
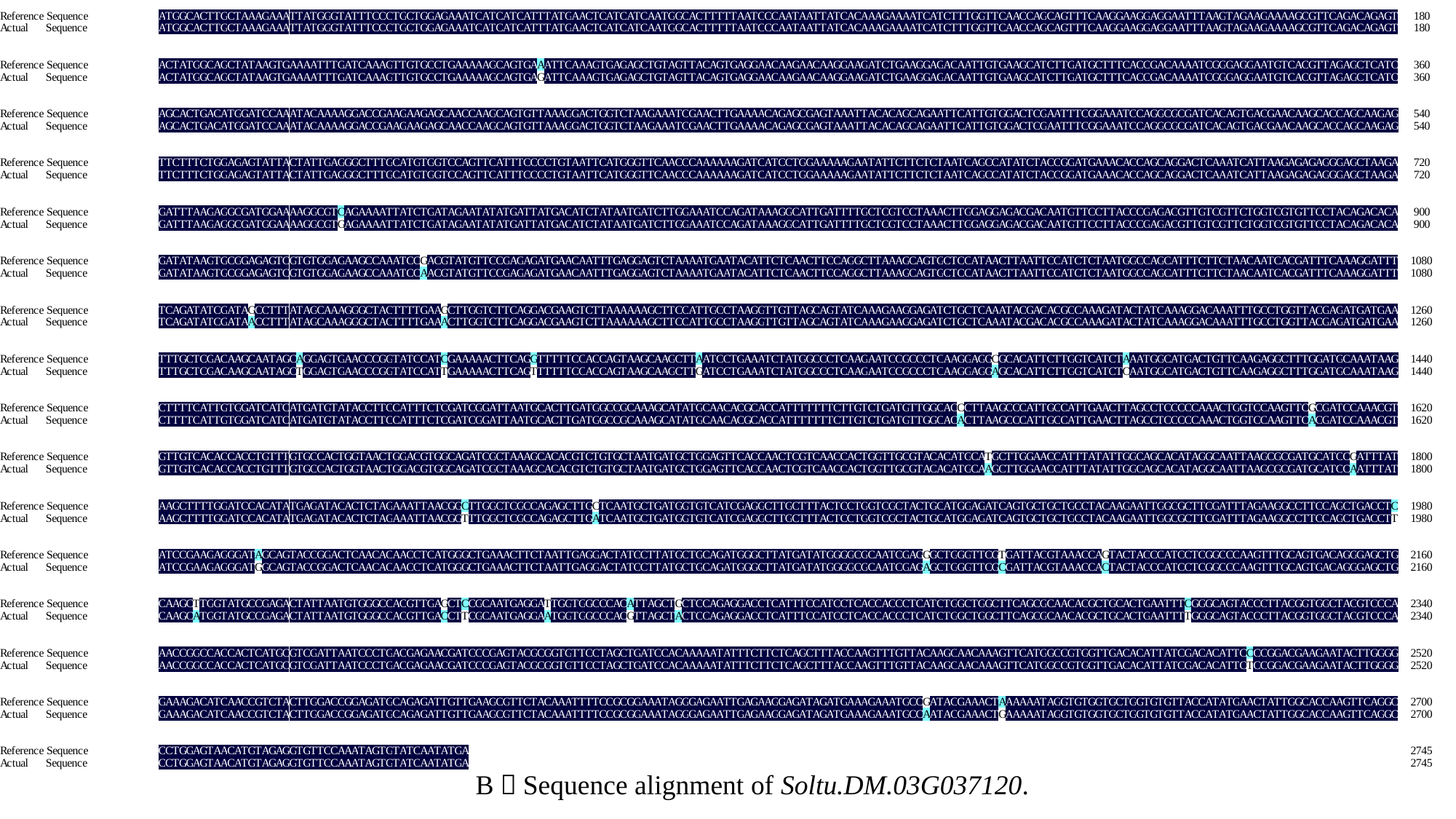

B：Sequence alignment of Soltu.DM.03G037120.

## Slide 3
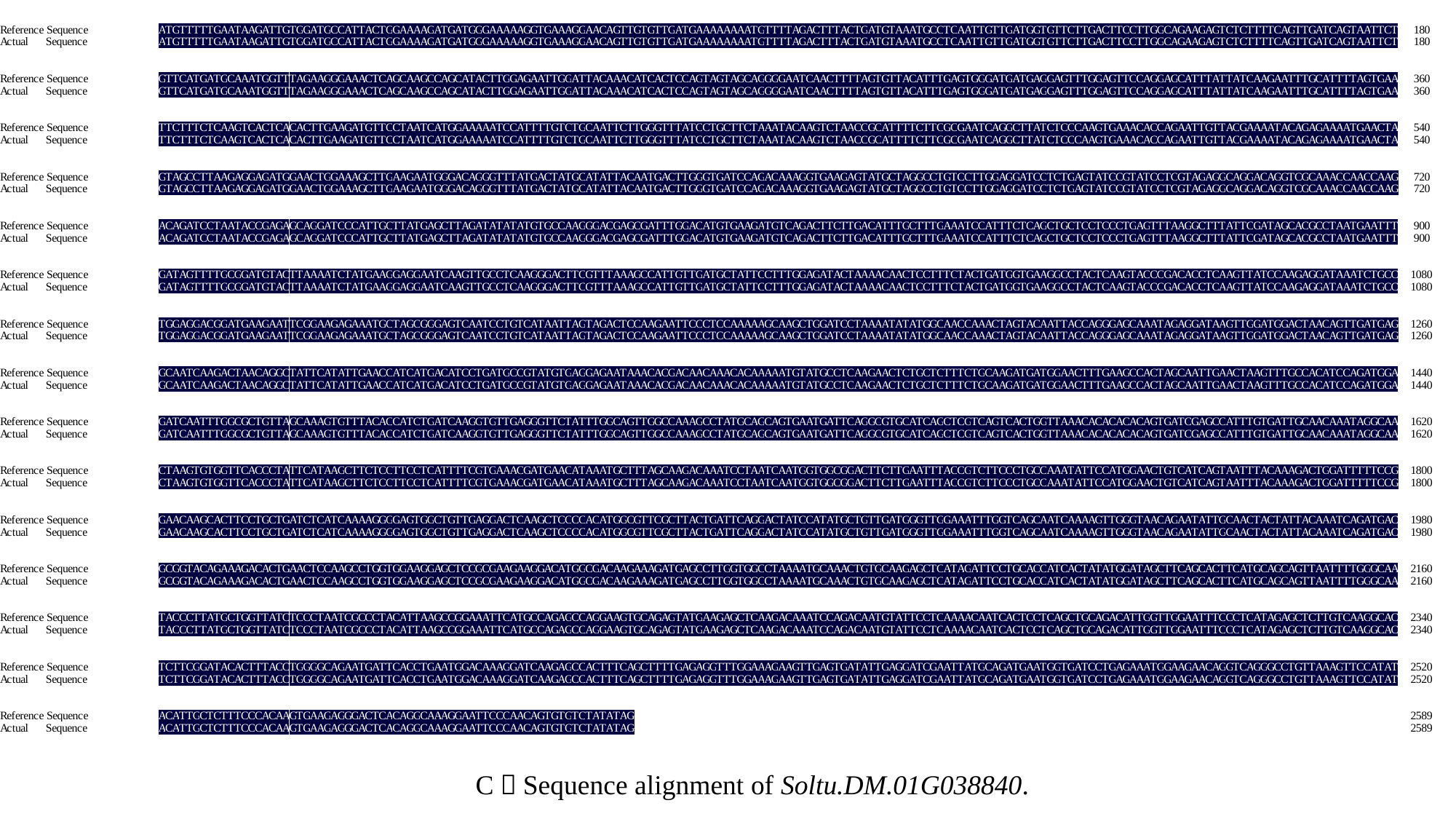

C：Sequence alignment of Soltu.DM.01G038840.
